# Glyoxalase 1 loss reduces fatty liver but impairs glucose handling in male mice

**DOI:** 10.14814/phy2.71106

**Published:** 2026-09-14

**Authors:** Emely A. Hoffman, Aiden M. Phoebe, Marissa N. Trujillo, Wei Chen Zhang, Erin Q. Jennings, Dominique O. Farrera, David J. Orlicky, Lauren N. Rutt, Rebecca L. McCullough, Andrea A. Huacachino, Mariola M. Marcinkiewicz, Nathaniel W. Snyder, Kassandra R. Bruner, Kimberly Bassoco Payan, Diego Juan Federico Martinez, Jennifer H. Stern, James J. Galligan

**Affiliations:** ^1^ Department of Pharmacology and Toxicology, College of Pharmacy University of Arizona Tucson Arizona USA; ^2^ Department of Pathology University of Colorado Anschutz Medical Campus Aurora Colorado USA; ^3^ Department of Pharmaceutical Sciences, the Skaggs School of Pharmacy and Pharmaceutical Sciences University of Colorado Anschutz Medical Campus Aurora Colorado USA; ^4^ Aging & Cardiovascular Discovery Center, Department of Cardiovascular Sciences Lewis Katz School of Medicine at Temple University Philadelphia Pennsylvania USA; ^5^ Division of Endocrinology, Department of Medicine, College of Medicine University of Arizona Tucson Arizona USA

**Keywords:** glycation, glycolysis, glyoxalase, MAFLD, methylglyoxal

## Abstract

We demonstrate the generation and characterization of the methylglyoxal detoxifying enzyme, glyoxalase 1, knockout mice (*Glo1* ^−/−^). With a 16‐week high‐fat high sucrose (HFHS) diet, *Glo1* ^−/−^ mice had a significant reduction of hepatic lipid accumulation when compared to WT counterparts. Paradoxically, these effects are limited to the liver, as no significant impact on white adipose tissue was observed. We also show that *Glo1* ^−/−^ mice have a significant worsening of glycemic control, showing a significant increase in fasting blood glucose and oral glucose tolerance. Although previous findings have suggested a role for GLO1 in kidney health, our results show no overt kidney phenotype resulting from Glo1 loss. Collectively, these results demonstrate a role for GLO1 in hepatic lipid metabolism while having a minimal impact on adipose or kidney function.

## Graphical abstract

Loss of glyoxalase 1 has a minimal impact on protein glycation in a murine model for metabolic syndrome. These mice have reduced hepatic steatosis despite impaired glucose handling.

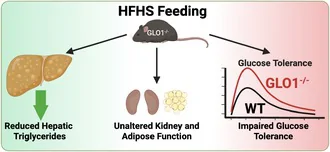

## 1 INTRODUCTION

Obesity is a worldwide pandemic and impacts nearly every organ in the body, including the liver. The prevalence of metabolic dysfunction‐associated fatty liver disease (MAFLD), caused by excessive hepatic adipose storage, is estimated to affect 24% of the US population (Organization, W.H, 2020; Younossi et al., 2018). MAFLD is present in greater than 70% of patients with type 2 diabetes (T2D), indicating MAFLD as a significant co‐morbidity in these individuals (Tilg et al., 2017; Williams et al., 2011). A major driving factor in the pathogenesis of obesity and MAFLD is the sustained consumption of high‐carbohydrate diets (Jensen et al., 2018); diets rich in fructose‐containing foods are capable of inducing insulin resistance in humans and metabolic syndrome in mice (Jensen et al., 2018). While the link between fructose consumption and MAFLD has been demonstrated, the precise mechanisms underlying this phenotype remain less understood.

Glycolysis is a tightly regulated metabolic pathway, ripe with feedback mechanisms to control flux and output (Li et al., 2019; Moellering & Cravatt, 2013; Tanner et al., 2018). Despite these checks, reactive by‐products, some of which prove deleterious to cell health, are generated (Galligan et al., 2018; Moellering & Cravatt, 2013). One such metabolite, methylglyoxal (MGO), is generated through the spontaneous degradation of dihydroxyacetone phosphate (DHAP), a triose phosphate (Sato et al., 1980). MGO concentrations represent ~0.1%–1% of the total glycolytic flux, yielding nM—μM concentrations (Galligan et al., 2018). Due to its electrophilic nature, MGO generates stable, long‐lived post‐translational modifications (PTMs) that are elevated in diabetic patients (Bierhaus et al., 2012; Galligan et al., 2018). To cope with this reactive metabolic by‐product, cells have a dedicated pathway, the glyoxalase cycle, to detoxify MGO (Rabbani et al., 2014). This two‐enzyme cycle is comprised of glyoxalase 1 (GLO1) and GLO2, converting MGO to lactate through the intermediate, lactoylglutathione (Farrera & Galligan, 2022). Reduced GLO1 expression has been reported in experimental models and patients with MAFLD; however, it is not known whether this is causal or compensatory (Spanos et al., 2018). Loss of function variants in GLO1 have been associated with diabetic nephropathy, although experimental studies have reached conflicting conclusions (Beisswenger et al., 2005; Bierhaus et al., 2012; Giacco et al., 2014; Qi et al., 2017; Wu et al., 2011). While Glo1 knockdown has been reported to promote diabetic kidney disease, complete genetic deletion failed to reproduce this phenotype, leaving the physiological role of GLO1 unresolved (Giacco et al., 2014; Schumacher et al., 2018).

To determine the role of GLO1 in obesity‐associated metabolic disease, we generated whole‐body Glo1 knockout (*Glo1* ^*−/−*^) mice and evaluated the effects of chronic high‐fat, high‐sucrose consumption on liver, adipose, kidney, and systemic glucose homeostasis. As reduced GLO1 expression has been reported in patients with MAFLD, we hypothesized that loss of GLO1 would exacerbate metabolic dysfunction by increasing MGO‐mediated stress.

## 2 EXPERIMENTAL METHODS

### 2.1 Animal studies

Male WT and *Glo1* ^−/−^ mice (8 weeks) were housed in specific pathogen‐free, temperature (68–70°F), humidity (30%–70%), and light (14 h on, 10 h off) controlled conditions (Gaffney et al., 2020). Mice were fed either a regular chow diet (NIH 7913, Envigo) or a high‐fat (36%) high sucrose (30%) diet (D11092103, Research Diets, Inc.) for 16 weeks. Food consumption and animal weights were measured weekly. At the completion of the study, mice were sacrificed via exsanguination followed by cervical dislocation. All animal use was approved by the Institutional Animal Care and Use Committee of the University of Arizona and adhered to the NIH Guide for the Care and Use of Laboratory Animals.

### 2.2 Generation of Glo1^−/−^ mice

*Glo1* ^*−/−*^ mice were created by CRISPR‐SpCas9 genome editing in the C57Bl/6NN strain through the University of Arizona Cancer Center Genetically Engineered Mouse (GEM). Exon 3 of the Glo1 gene was targeted with guide RNAs (gRNAs) designed using CRISPOR web tool as follows 5′CCGACTGCAAAGGCGTCCCCCGG and 5′ACACGCTCCACAAGGCCCGGGGG for the 5′ double‐stranded break and 3′AAAGTGCCTGCCCGTCTAAAGGG and 3′ GAAAGTGCCTGCCCGTCTAAAGG for the 3′ double‐stranded break (Synthego). Ribonucleoprotein complexes were assembled with the gRNAs and Alt‐R S.p. HiFi Cas9 Nuclease V3 recombinant protein prior to (Integrated DNA Technologies) microinjection into the pronucleus of 2 cell C57Bl/6NJ zygotes. Pups were screened by polymerase chain reaction (PCR) for evidence of frameshift deletions produced by non‐homologous end joining. Breeding was initially set up as trios (F1: 1 male and 2 females) of a *Glo1* heterozygous founder mouse to WT. Breeding was moved to heterozygous pairs and maintained continuously. Genotyping was performed using these primer sequences: *Glo1* Exon 3 ACTCAGCCGACTGCAAAGG and GAGCAACCGCAGTAGTTTGG. WT animals were confirmed through visualization of the *Glo1* PCR amplicon at 639 bp. Knockout animals were confirmed by the presence of a large deletion, observed as a band at 150 bp. Heterozygotes were confirmed if both bands were present.

### 2.3 Glucose tolerance tests

After 15 weeks on study, mice (*N* = 5–9 per cohort) were fasted for 6 h and then given 2 g/kg glucose by oral gavage. Blood was collected via tail vein at 0 (immediately prior to injection), 15, 30, 60, 90, and 120 min after glucose injection. Blood glucose was determined at each time point using an Accu‐check glucometer and plotted over time. Area under the curve was determined using GraphPad Prism Version 10.2.

### 2.4 Insulin tolerance tests

72 h following the glucose tolerance testing, mice (*N* = 5–7 per cohort) were again fasted for 6 h and then injected intraperitoneally with 1 U/kg *Humulin R* (Eli Lilly, HI‐210). Blood was collected from a tail vein at 0 (immediately prior to injection), 15, 30, 60, 90, and 120 min after treatment. Blood glucose was determined at each time point using an Accu‐check glucometer and % suppression was plotted over time. The glucose disappearance rate (kITT) was determined based on the rate of blood glucose decrease using the ITT data. The lowest glucose level reached was subtracted from the initial glucose value and divided by the time to reach that value. Data is presented as the percent decline per minute for each mouse.

### 2.5 Histology

Liver and kidney were excised, sectioned, and immediately fixed in 10% neutral buffered formalin (MilliporeSigma) for 24 h. Tissues were then washed twice with PBS followed by immersion in 70% ethanol. Tissues were dehydrated, embedded in paraffin, cut, and sections mounted on slides. Tissues were stained with hematoxylin and eosin (H&E).

### 2.6 Tissue injury scoring

Liver damage was scored as previously described (Lanaspa et al., 2018). Briefly, the extent of steatosis, the presence of inflammatory cells and foci, features of hepatocyte injury, and markers of tissue response was determined by cell morphology and staining by a trained histopathologist; an entire section of liver from all 5–7 mice/group was examined and the extent of each criterion was scored by the histopathologist. Then, to assess fibrotic changes, Picro‐Sirius Red (Sigma‐Aldrich, 365548) stained sections (*N* = 5 per slide) were examined using polarized light, and positive pixels and total pixels in each of 5, 40× images were quantified using the 3I Slidebook program (3I, Intelligent Imaging Innovations, Denver, Colorado) and an average percent positive pixel was calculated for each slide (Shearn et al., 2017). Histologic images were captured on an Olympus BX51 microscope equipped with a 4MP Macrofire digital camera (Optronics) using the PictureFrame Application 2.3 (Optronics). All images were cropped and assembled using Photoshop CS2 (Adobe Systems, Inc.).

### 2.7 Adipocyte area calculation

Adipocyte area was calculated using ImageJ with the Adiposoft plugin (Galarraga et al., 2012). Three images per mouse were analyzed to quantify total adipocyte area. The average of each image was then used to calculate an average of averages per mouse. Total pixels were calculated using the scale bar (50 μm), yielding 0.467 μm per pixel. Data is plotted as μm (Younossi et al., 2018).

### 2.8 Serum ALT

After 16 weeks on study, nonfasted mice were harvested and blood was collected via cardiac puncture and placed into heparinized tubes. Serum was isolated through centrifugation 3000 rpm for 10 mins. Alanine aminotransferase (ALT) was quantified from 14 μL serum using a commercially available kit (Cat. No. 31830, Sekisui Diagnostics) as previously described (Harris et al., 2015).

### 2.9 Immunohistochemistry

Paraffin‐embedded liver sections were deparaffinized and stained with an antibody against glyoxalase 1 (GLO1, catalog no. 15140‐1‐AP; Proteintech, Rosemont, IL). Immunohistochemistry images were acquired using a brightfield microscope (Zeiss Microsystems). Formalin samples are coded at the time of collection for a blinded analysis; at least three images were acquired per tissue section. No specific immunostaining was seen in sections incubated with PBS rather than the primary antibody (data not shown).

### 2.10 Serum leptin

After 16 weeks on study, nonfasted mice were harvested and blood was collected via cardiac puncture and placed into heparinized tubes. Serum was isolated through centrifugation at 3000 rpm for 10 mins. Serum leptin was measured using 5 μL serum in 95 μL provided diluent and a standard curve spanning 0–8 ng with a commercially available ELISA kit (Cat. No. DY498‐05, R&D Systems).

### 2.11 Serum insulin

Insulin was quantified from 5 μL of flash frozen serum using an ultrasensitive ELISA from ALPCO (80‐INSMSU‐E01) according to the manufacturer's directions. The concentration was calculated in ng/mL via a 5 parameter curve.

### 2.12 Triglycerides

Hepatic and serum triglycerides were determined using a commercially available kit (Cat. No. 23660, Sekisui Diagnostics) according to the manufacturer's protocol. 450 μL of ice‐cold PBS was added to ~50 mg of tissue and samples were sonicated at 40% power, for 2 rounds of 10 pulses. 2.25 mL of 2:1 chloroform: methanol was then added and samples were briefly vortexed prior to the addition of 1 mL of ddH_2_O. Samples were then centrifuged for 5 min at 2200 × *g*. Extracted lipids (organic layer) were dried under N_2_ and resuspended in 800 μL of ethanol. The standard curve ranged from 1000 mg/dL (11.3 mmol/L) to 0 mg/dL. Tissue samples were diluted 1:160 and 5 μL were plated. Absorbance was read at 515 nm and concentrations were determined. Serum triglycerides were diluted 1:12.5 in ddH_2_O. Samples were diluted 1:16 and triglyceride concentrations were determined as described.

### 2.13 Serum creatinine

Creatinine was quantified from 2 μL of serum using a commercially available assay (Cat. No. 22130, Sekisui Diagnostics) according to the manufacturer's protocol. The standard curve ranged from 4 mg/dL (354 μmol/L) to 0 mg/dL. Serum was diluted 1:50 in the enzyme mixture, and kinetic absorbance was read at 510 nm.

### 2.14 Kidney glycogen

Kidney glycogen content was assayed as previously described (Bruner et al., 2025; Marx et al., 2026) and according to the method of Lo et al (Lo et al., 1970). Briefly, kidneys were first powdered using a liquid nitrogen cooled mortar and pestle. Frozen powdered kidney was then weighed immediately prior to boiling in 30% KOH saturated with NA_2_SO_4_ for 30 min. Glycogen was next precipitated with 95% ethanol, followed by centrifugation at 3000 × *g* for 30 min. Supernatant was then removed and glycogen pellets were isolated and dissolved in distilled H2O, followed by the addition of 5% phenol. H_2_SO_4_ was then forcefully pipetted into the sample within a borosilicate glass tube. Finally, samples were incubated 30°C for 20 min, then transferred to a 96 well plate, and read at 490 nm.

### 2.15 Serum blood urea nitrogen (BUN)

BUN was measured using a commercially available kit (Cat. No. 28330, Sekisui Diagnostics, Charlottetown, PE, Canada). Urea concentrations were assessed according to the manufacturer's protocol. The standard curve ranged from 0 mg/dL to 140 mg/dL (50 mmol/L). Serum was diluted 1:99 in the enzyme mixture. The plate was read at 340 nm for 180 s with 15 s intervals after a 30 min incubation at 37°C.

### 2.16 MGO quantification

300 μL of 80:20 MeOH:ddH_2_O with 0.1% acetic acid v/v (−80°C) containing 50 pmol ^13^C_3_‐MGO (synthesized in‐house, see (Galligan et al., 2018)) was added to ~15 mg of tissue. Tissues were homogenized using a bead mill homogenizer (Omni International) and insoluble protein was removed via centrifugation at 14,000 × *g* for 10 min at 4°C. 100 μL of supernatant was used for MGO quantification and derivatized with 10 μL 500 μM 4‐methoxy‐*o*‐phenylenediamine (AmBeed) in a 37°C incubator overnight. For serum samples, 10 μL of serum was added to 190 μL of 80:20 MeOH:ddH_2_O with 0.1% acetic acid (−80°C) containing 50 pmol ^13^C_3_‐MGO. Insoluble protein was removed via centrifugation at 14,000 × *g* for 10 min at 4°C. Derivatized samples were centrifuged at 14,000 × *g* for 10 min and the supernatant was chromatographed using a Shimadzu LC system equipped with a 50 × 2.1 mm, 3 μm particle diameter Atlantis C_18_ column (Waters) at a flow rate of 0.5 mL/min. Buffer A (0.1% formic acid in H_2_O) was held at 95% for 1 min then a linear gradient to 80% solvent B (0.1% formic acid in MeOH) was applied over 2 min, followed by a linear gradient to 100% solvent B over 2 min. The column was held at 100% B for 1 min and then washed and equilibrated at 99% A for 2 min. Multiple reaction monitoring (MRM) was conducted in positive ion mode using an AB SCIEX 6500 QTRAP+ with the following transitions: *m/z* 175.0 → 160.0 (analyte); *m/z* 178.0 → 163.0 (^13^C_3_‐MGO, internal standard).

### 2.17 GSH and LGSH quantification

300 μL of 80:20 MeOH:ddH_2_O with 0.1% formic acid v/v (−80°C) containing 1.5 nmol ^13^C‐GSH was added to ~15 mg of tissue. Tissues were homogenized using a bead mill homogenizer (Omni International). Insoluble protein was removed via centrifugation at 14,000 × *g* for 10 min at 4°C. Samples were dried using a Savant SpeedVac (ThermoScientific) until no liquid remained, then resuspended with 100 μL of 80:20 MeOH:ddH_2_O with 0.1% formic acid v/v. Tubes were sonicated briefly to dislodge the pellet. Debris was removed via centrifugation at 14,000 × *g* for 10 min, and samples were chromatographed using a Shimadzu LC system equipped with a 100 × 3 mm, 3 μm particle diameter Luna C18(2) column (Phenomenex) at a flow rate of 0.5 mL/min. Buffer A (10 mM HFBA in H_2_O) was held at 95% for 1 min, then a linear gradient to 75% buffer A was applied over 2 min. This was held for 3 min before a linear gradient was applied to reach 50% buffer A over 3 min. This was held for 3 min before a linear gradient was applied to reach 98% solvent B (10 mM HFBA in acetonitrile), which was then held for 3 min before washing and equilibrating with 95% buffer A for 2 min. Multiple reaction monitoring (MRM) was conducted in positive ion mode using an AB SCIEX 6500 QTRAP+ with the following transitions: *m/z* 380.1 → 233.1 (LGSH); *m/z* 308.0 → 179.0 (GSH); *m/z* 311.0 → 182.0 (^13^C‐GSH, internal standard).

### 2.18 Quantitation of PTMs

Lysis buffer containing 100 mM Tris–HCl, 150 mM NaCl, 10 mM NaF, 1% Triton X‐100, pH = 7.4 with 1:500 protease/phosphatase inhibitor cocktail (Sigma, P8340 and P5726) was added to tissue samples weighing ~15 mg (liver and kidney). Protein was quantified using BCA assay (ThermoFisher, 23,227). 200 μg of protein was aliquoted and precipitated using acetone overnight at −20°C followed by centrifugation at 14,000 × *g* for 10 min. Pellets were resuspended in 65 μL 50 mM ammonium bicarbonate and spiked with 10 μL of a master mix containing internal standards of interest. Proteins were digested via the addition of 10 μL of sequencing grade trypsin (0.1 mg/mL) (Promega, V5111) for 6 h at 37°C. Trypsin was denatured via boiling at 95°C for 10 min, and samples were cooled to room temperature. Aminopeptidase (15 μg in 10 μL, EMD Millipore, 164,598) was added to each sample and incubated overnight at 37°C. Aminopeptidase was denatured via heating at 95°C for 10 min, and samples were cooled to room temperature. 1:1 HFBA:H_2_O (15 μL) was added to each sample. Debris were removed via centrifugation at 14,000 × *g* for 10 min and samples were chromatographed using a Shimadzu LC system equipped with a 150 × 2.1 mm, 3.5 μm particle diameter Eclipse XDB‐C8 column (Agilent) at a flow rate of 0.5 mL/min. Buffer A (10 mM HFBA in H_2_O) was held at 99% for 1 min, then a linear gradient was applied over 8 min to 50% buffer A, followed by a 2 min linear gradient to reach 98% solvent B (10 mM HFBA in acetonitrile). This was held for 3.5 min before washing and equilibrating in 99% buffer A for 3 min. Scheduled MRM was conducted in positive mode using an AB SCIEX 6500 QTRAP+. The following parameters (Table 1) were used for detection and as previously described (Gaffney et al., 2020; Galligan et al., 2017):

**TABLE 1:** MS parameters used for PTM quantification.

Species	Q1 (*m/z*)	Q3 (*m/z*)	Time (min)	CE (V)
Lys	147.0	84.0	3.9	25
^13^C_6_^15^N_2_ Lys	155.0	90.0	3.9	25
Arg	175.0	70.0	4.6	51
^13^C_6_^15^N_4_ Arg	185.0	75.0	4.6	51
Leu	132.0	86.0	6.0	14
^13^C_6_^15^N Leu	138.0	91.0	6.0	14
MG‐H1	229.2	70.0	5.3	42
^13^C‐MG‐H1	230.2	70.0	5.3	42
CEA	247.2	70.0	5.2	47
^13^C‐CEA	248.2	70.0	5.2	47
LactoylLys	219.2	84.0	3.2	29
CEL	219.2	84.0	4.0	27
CEL‐d_4_	223.2	88.0	4.0	27

### 2.19 SDS‐page

Liver samples (~10 mg) were homogenized in 300 μL of a buffer containing 150 mM NaCl, 50 mM HEPES, and 1% IGEPAL, pH 7.4. Samples were briefly sonicated (10 × 1 s pulses) and centrifuged at 14,000 × *g* for 10 min at 4°C. Soluble protein was then resolved as previously described (Gaffney et al., 2020). Antibodies targeting GLO1 (1:1000, Proteintech, 15,140‐1‐AP), phospho‐AKT Ser473 (1:2000, Cell Signaling Technology, #9271S), AKT (1:2000, Cell Signaling Technology, #9272S), phospho‐insulin receptor (1:2000, Cell Signaling Technology, #3023S), insulin receptor (1:2000, Cell Signaling Technology, #3025S), and β‐actin (1:1000, Sigma, A1978) were incubated with membranes overnight at 4°C. The following day, membranes were rinsed 3X with TBST and incubated with infrared secondary antibody (1:5000, LICORbio, 926‐32213 and 926‐68022) for 45 min at RT. After rinsing 3X with TBST, membranes were imaged using c600 Azure imaging system. Images were quantified using ImageJ. Phosphorylation (e.g. pAKTS473) was normalized to actin and then normalized to the actin normalized total protein (e.g. AKT).

### 2.20 Statistical analysis

Data were quantified using a two‐way ANOVA using a Tukey post‐hoc test. Differences were significant when *p* < 0.05 with the *N* indicated in each figure legend. All analyses were carried out using Prism 10 for Macintosh, GraphPad Software.

## 3 RESULTS

### 3.1 GLO1 plays a role in systemic glucose control independent of insulin activity in obese mice

**FIGURE 1:**
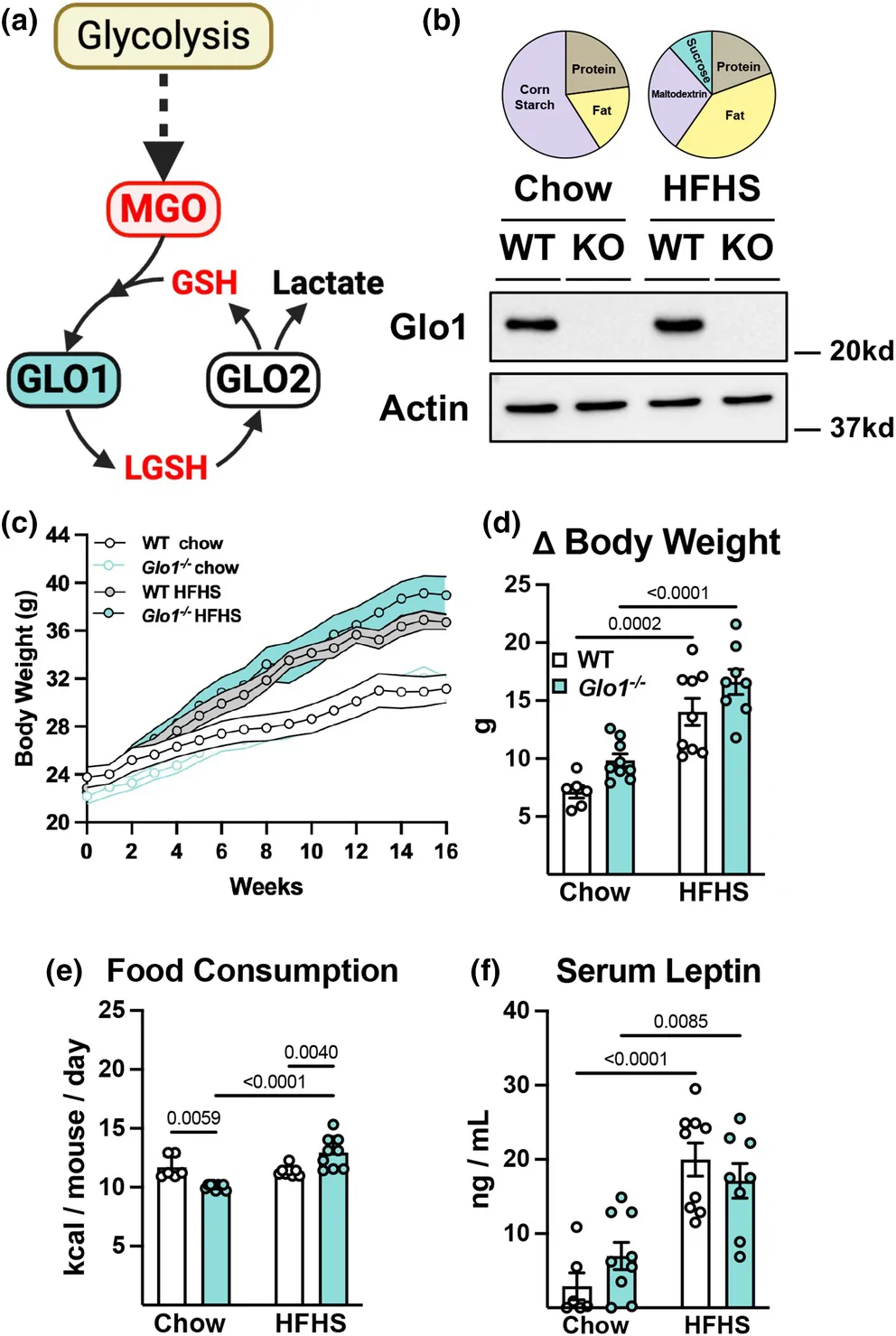
Loss of GLO1 does not significantly affect body weight gain in mice fed a high‐fat high‐sucrose diet when compared to WT mice. (a) The glyoxalase cycle. Created with BioRender.com. (b) Diet composition and western blot confirmation of WT and *Glo1* ^−/−^ mice genotypes. (c) Body weight was measured once per week. (d) Both WT and *Glo1* ^−/−^ mice display a significant increase in body weight, consistent with HFHS feeding. (e) Food consumption was also measured by weighing food weekly. (f) Serum leptin showed significant increase with HFHS feeding, but no genotype differences at 16 weeks. Chow‐fed WT (*N* = 6), chow‐fed *Glo1* ^−/−^ mice (*N* = 9), HFHS‐fed WT mice (*N* = 9), HFHS‐fed *Glo1* ^−/−^ mice (*N* = 8). Statistical significance was determined via Two‐Way ANOVA.

GLO1 detoxifies the reactive aldehyde glycolytic by‐product MGO (Figure 1a). We and others have shown that hyperglycemia results in a concomitant increase in MGO (Galligan et al., 2018; Queisser et al., 2010). To evaluate the role of GLO1 in the pathogenesis of diabetes, we generated full‐body *Glo1* ^*−/−*^ mice using CRISPR‐Cas9; these mice displayed no detectable *Glo1* expression Figure 1b. We then fed wild‐type (WT) or *Glo1* ^−/−^ mice either a standard chow or high fat high sucrose (HFHS) diet for 16 weeks, which results in fatty liver disease and reduced glucose tolerance (Ishimoto et al., 2013). At 16 weeks, *Glo1* expression remains unchanged in WT mice on either diet. As is consistent with a HFHS diet, WT mice display a significant increase in body weight; however, no differences are observed between WT and *Glo1* ^−/−^ mice (Figure 1c,d). This lack of a change in body weight between the genotypes is particularly interesting given the observed increase in HFHS consumption by the *Glo1* ^−/−^ mice (Figure 1e), which is independent of serum leptin concentrations (Figure 1f).

**FIGURE 2:**
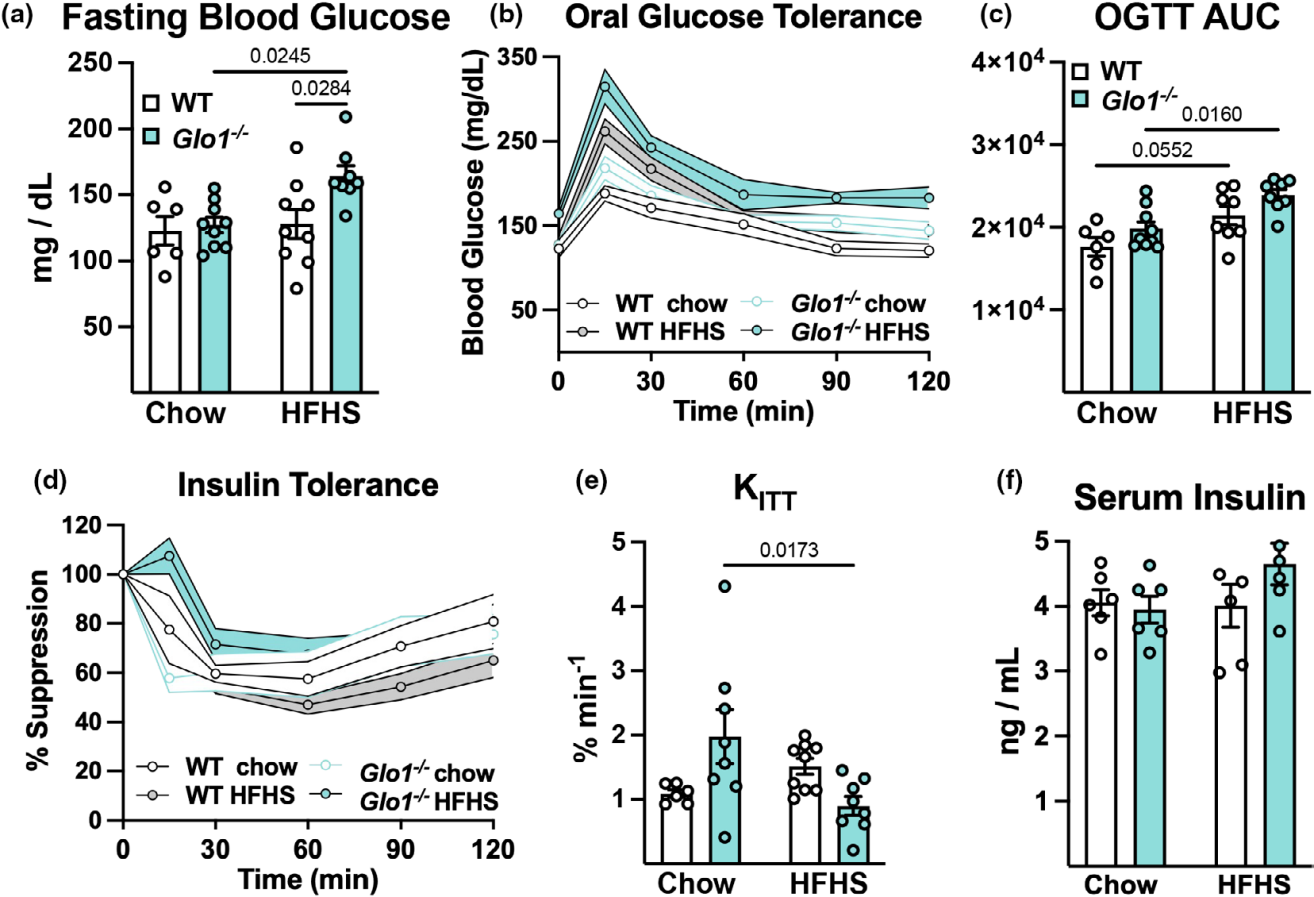
Obese GLO1 knockout mice have exacerbated glucose intolerance. (a) Fasting blood glucose is significantly elevated in *Glo1* ^−/−^ HFHS‐fed mice at 16 weeks. (b, c) Oral glucose tolerance testing (OGTT) and area under the curve (AUC) show a diet effect in both cohorts. (d) Insulin tolerance testing (ITT) shows no diet or genotype effect; however, (e) the constant of glucose decay shows a significant reduction in HFHS‐fed *Glo1* ^*−/−*^ mice. Lastly, (f) serum insulin remains unaffected in all cohorts. Chow‐fed WT (*N* = 6), chow‐fed *Glo1* ^−/−^ mice (*N* = 9), HFHS‐fed WT mice (*N* = 9), HFHS‐fed *Glo1* ^−/−^ mice (*N* = 8). Statistical significance was determined via Two‐Way ANOVA.

As *glo1* ^*−/−*^ zebrafish have been shown to have increased fasting blood glucose following 8 weeks of over‐feeding, we sought to evaluate glycemic control in our model (Lodd et al., 2019). As shown in Figure 2a, *Glo1* ^−/−^ mice have a significant increase in their fasting blood glucose. We next quantified glucose tolerance using an oral glucose tolerance test (OGTT), revealing a significant reduction in the ability of *Glo1* ^−/−^ mice to clear systemic glucose when compared to chow‐fed counterparts; however, no significant differences were observed when comparing HFHS‐fed WT and *Glo1* ^*−/−*^ mice (Figure 2b,c). An insulin tolerance test, however, found no significant differences between any of the cohorts (Figure 2d). A calculation of the constant of glucose decay (kITT), however, reveals a significant reduction in insulin responsiveness in HFHS‐fed *Glo1* ^*−/−*^ mice (Figure 2e). We also quantified serum insulin in each cohort (Figure 2f), revealing no significant differences resulting from either diet or genotype. Lastly, we evaluated insulin signaling via phosphorylation of insulin receptor and AKT (Ser473) (Figure S1). Consistent with the ITT and serum insulin levels, no significant differences were observed resulting from either diet or genotype. A key caveat, however, is that these samples were collected from mice with access to food (i.e. not fasted) and thus, we do not know how GLO1 impacts glucose stimulated insulin secretion. Collectively, these findings suggest that Glo1 deficiency alters glucose homeostasis in a diet‐dependent manner, with improved insulin responsiveness under chow conditions but impaired adaptation to HFHS feeding.

### 3.2 GLO1 has a minimal role on adipose biology in vivo

**FIGURE 3:**
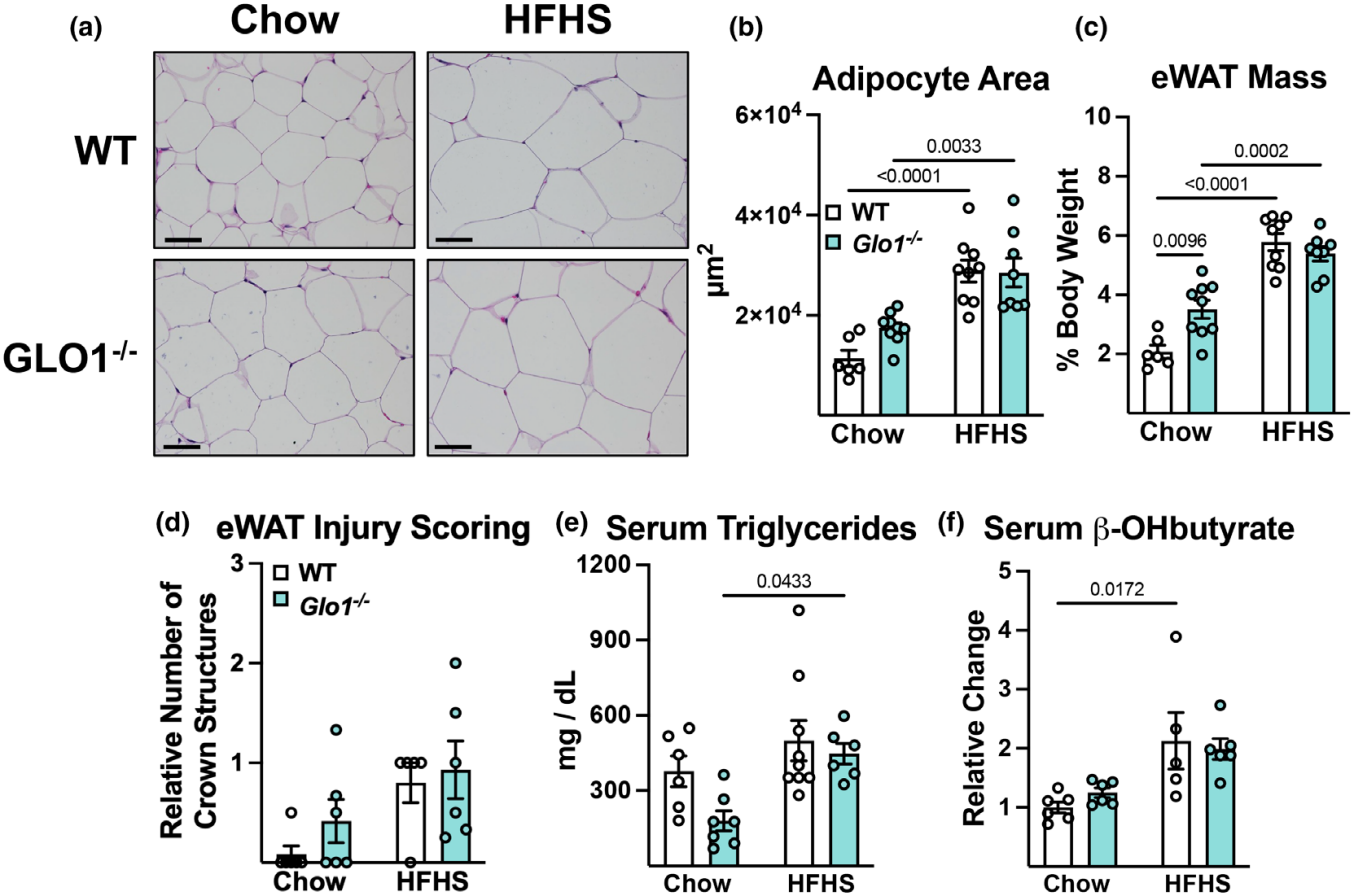
*GLO1* ^−/−^ does not alter eWAT mass nor adipocyte size in vivo. (a) Representative images of H&E stained eWAT. Scale bar represents 50 μm. (b) Adipocyte area was quantified using the Adiposoft plugin for imageJ, revealing a significant increase in adipocyte size in the HFHS‐fed cohort. No significant differences were observed in *Glo1* ^−/−^ mice. (c) eWAT mass is significantly increased in chow‐fed *Glo1* ^−/−^ mice, while no significant differences were observed in the HFHS‐fed cohorts. (d) Crown structures were quantified as a measure of eWAT injury, showing no significant differences in any cohort. *N* = 6 for all cohorts. (e) Serum triglycerides are significantly elevated in *Glo1* ^−/−^ when comparing the diet effect; however, no change was observed in WT mice. (f) As a measure of fatty acid oxidation, serum β‐hydroxybutyrate was measured, revealing a significant increase in WT mice with no effects observed in *Glo1* ^−/−^ mice. Unless otherwise stated: Chow‐fed WT (*N* = 6), chow‐fed *Glo1* ^−/−^ mice (*N* = 9), HFHS‐fed WT mice (*N* = 9), HFHS‐fed *Glo1* ^−/−^ mice (*N* = 8). Statistical significance was determined via Two‐Way ANOVA.

We recently published that cultured NIH3T3 fibroblasts fail to differentiate to adipocytes in the absence of GLO1 (Trujillo et al., 2025). As a logical extension, we evaluated epididymal white adipose tissue (eWAT) in this model. Not surprisingly, 16 weeks of a HFHS diet results in a significant increase in adipocyte area (Figure 3a,b) and eWAT mass (Figure 3c and Figure S2). Surprisingly, *Glo1* ^−/−^ mice have an increase in eWAT mass when fed a standard chow diet; however, no significant differences are observed between WT and *Glo1* ^−/−^ when fed a HFHS diet. We next counted crown structures as a marker for eWAT injury, revealing no significant differences between WT and *Glo1* ^−/−^ mice on either diet (Figure 3d). Lastly, we reasoned that if *Glo1* ^−/−^ mice have increased triglyceride export or elevated fatty acid oxidation, a quantification of serum triglycerides (Figure 3e) and serum β‐hydroxybutyrate (Figure 3f) would reveal differences between WT and *Glo1* ^−/−^ mice. However, this is not the case, as serum triglycerides and β‐hydroxybutyrate only vary by diet but not genotype.

### 3.3 Hepatic triglycerides are reduced in *Glo1* ^−/−^ mice

**FIGURE 4:**
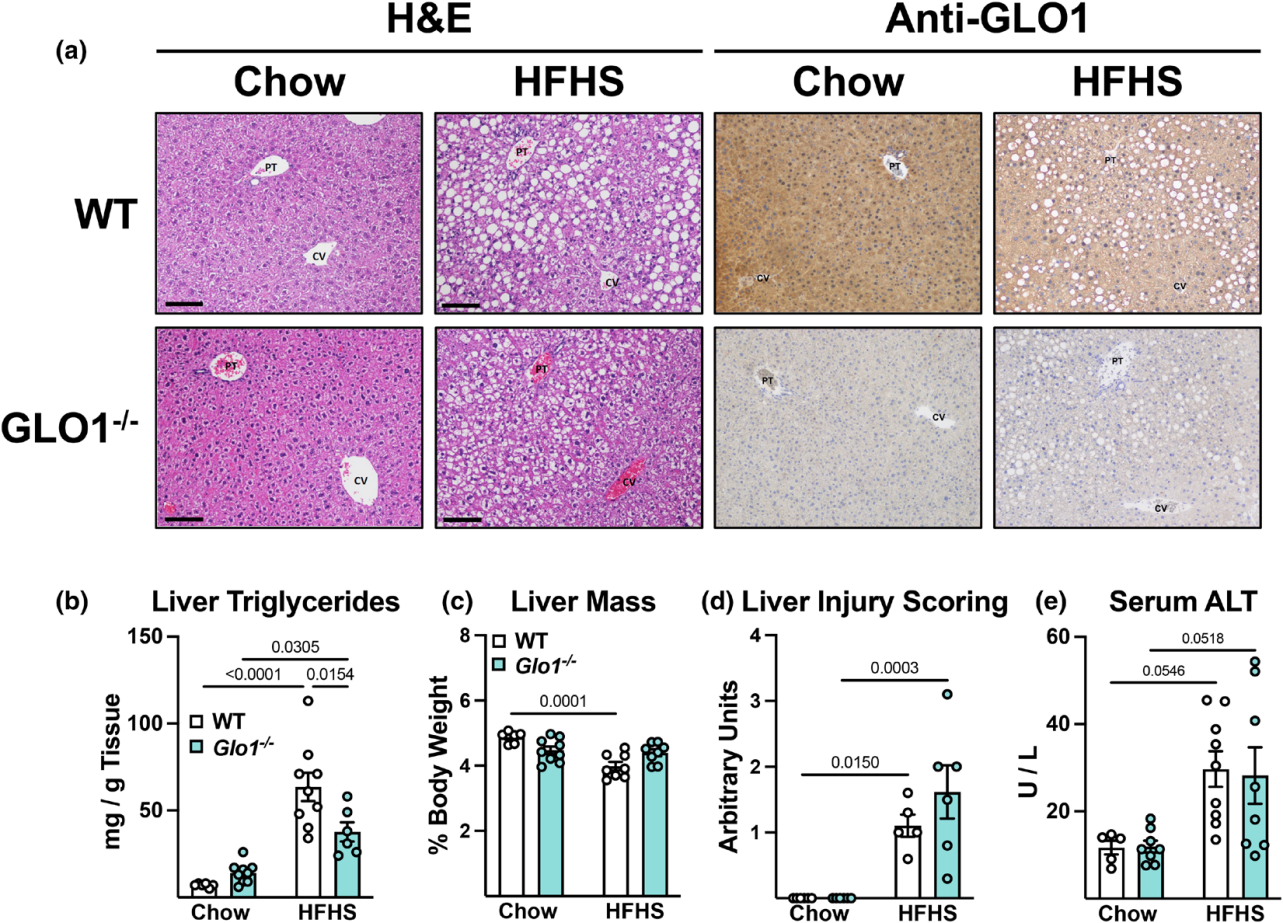
*Glo1* ^−/−^ mice have reduced hepatic triglyceride accumulation in response to HFHS feeding. (a) Representative images of H&E‐stained liver sections. Immunohistochemistry for GLO1 in each cohort shows no ubiquitous expression in WT mice. CV, central vein; PT, portal vein. Scale bar indicates 100 μm. (b) Hepatic triglyceride levels are elevated in HFHS‐fed mice, though significantly less so in *Glo1* ^−/−^. (c) Liver mass to body weight ratio is unchanged in *Glo1* ^−/−^ mice. (d) Liver injury scoring is significantly elevated in HFHS‐fed mice. *N* = 6 for all cohorts. (e) Serum alanine aminotransferase (ALT) levels are elevated, although not significantly, in HFHS‐fed mice. Unless otherwise stated: Chow‐fed WT (*N* = 6), chow‐fed *Glo1* ^−/−^ mice (*N* = 9), HFHS‐fed WT mice (*N* = 9), HFHS‐fed *Glo1* ^−/−^ mice (*N* = 8). Statistical significance was determined via Two‐Way ANOVA.

Diets rich in fat and fructose are well‐documented to result in MAFLD in rodents and humans (Ishimoto et al., 2013; Kim et al., 2017; Lally et al., 2019; Softic et al., 2019). We thus reasoned that while no notable differences in eWAT are found between WT and *Glo1* ^−/−^, perhaps hepatic lipid homeostasis would be disrupted. Consistent with previous reports, our feeding regimen results in substantial hepatic steatosis in WT HFHS‐fed mice (Figure 4a,b). These effects, however, are modestly blunted in HFHS‐fed *Glo1* ^−/−^ mice with no significant differences found between WT and *Glo1* ^−/−^ mice on a chow‐fed diet. Interestingly, despite a significant rise in hepatic triglycerides, WT mice display a significant reduction in liver mass to body weight ratio when fed a HFHS diet, while *Glo1* ^−/−^ mice display no significant differences in either feeding regimen (Figure 4c and Figure S2). Similarly, quantification of liver injury and serum alanine aminotransferase (ALT), a marker for liver damage, shows both *Glo1* ^−/−^ and WT mice display a significant increase in liver injury scoring when fed a HFHS diet, although this is largely a result of hepatocyte lipid accumulation as injury (Figure 4d,e) (Orlicky et al., 2019).

**FIGURE 5:**
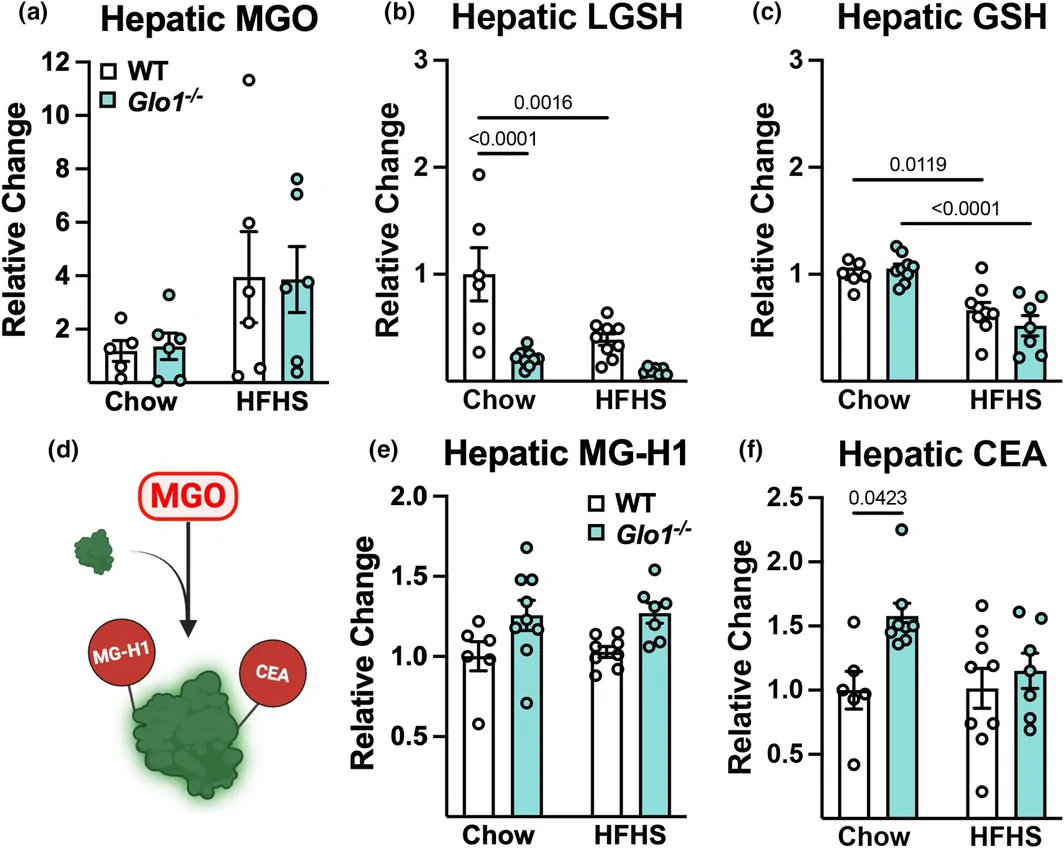
Hepatic MGO and MGO‐derived PTMs are largely unchanged in *Glo1* ^−/−^ mice. (a) Hepatic MGO is unchanged in all cohorts. *N* = 6 for all cohorts. (b) Consistent with a lack of GLO1 activity, LGSH is significantly decreased in chow‐fed *Glo1* ^−/−^ mice. HFHS feeding results in a significant decrease in hepatic LGSH and (c) total GSH. (d) MGO modifies cellular proteins to yield the PTMs, MGO‐hydroimidazolone 1 (MG‐H1) and carboxyethylarginine (CEA). Created with BioRender.com. (e) Hepatic MG‐H1 is significantly reduced in WT HFHS‐fed mice, while *Glo1* ^−/−^ mice have elevated MG‐H1 when comparing HFHS‐fed cohorts. (f) No significant differences are observed in hepatic CEA. Unless otherwise stated: Chow‐fed WT (*N* = 6), chow‐fed *Glo1* ^−/−^ mice (*N* = 9), HFHS‐fed WT mice (*N* = 9), HFHS‐fed *Glo1* ^−/−^ mice (*N* = 8). Statistical significance was determined via Two‐Way ANOVA.

As shown in Figure 1a, GLO1 metabolizes MGO into the acylglutathione intermediate, lactoylglutathione, LGSH (Farrera & Galligan, 2022). We and others have shown that under conditions of reduced GLO1 activity, the basal levels of MGO and MGO‐derived PTMs are unchanged (Galligan et al., 2018; Schumacher et al., 2018; Trujillo et al., 2025); however, when challenged with either hyperglycemia or exogenous MGO, intracellular MGO and concomitant PTMs increase (Gaffney et al., 2020; Galligan et al., 2018; Trujillo et al., 2025). To evaluate this in the context of metabolic syndrome, we quantified hepatic MGO (Galligan et al., 2018; Rabbani & Thornalley, 2014). As shown in Figure 5a, free MGO is not significantly elevated among any of the treatment groups. Importantly, the product of GLO1 activity, LGSH, is significantly reduced in *Glo1* ^−/−^ mice, consistent with a loss of activity Figure 5b. An important consideration regarding LGSH is that the intracellular concentrations are ~3–5 orders of magnitude lower than GSH, which is required for GLO1 activity. As shown in Figure 5c, a marked diet‐induced reduction in GSH is observed, regardless of genotype. Thus, we cannot rule out that the significant reduction in LGSH is not, at least in part, due to the reduction in GSH, but the *Glo1* ^*−/−*^ effect remains significant.

Due to its reactive nature, we hypothesized that elevations in MGO may be observed as PTMs, rather than the free aldehyde (Figure 5d). We thus quantified MGO‐derived PTMs using a highly sensitive MS‐based approach (Galligan et al., 2017, 2018). While the levels of MGO‐hydroimidazolone 1 (MG‐H1) are not significantly affected by diet or genotype (Figure 5e), the levels of carboxyethylArg (CEA) are significantly elevated in *Glo1* ^*−/−*^ chow‐fed mice, while no changes in this PTM are observed resulting from a HFHS diet (Figure 5f). Collectively, our data contradict previous reports indicating significant elevations in MGO‐derived modifications resulting from diet‐induced metabolic disorder(s) (Kondanna et al., 2026; Lai et al., 2024; Lopez Gonzalez et al., 2025).

### 3.4 Ablation of GLO1 is not sufficient to drive diabetic nephropathy

**FIGURE 6:**
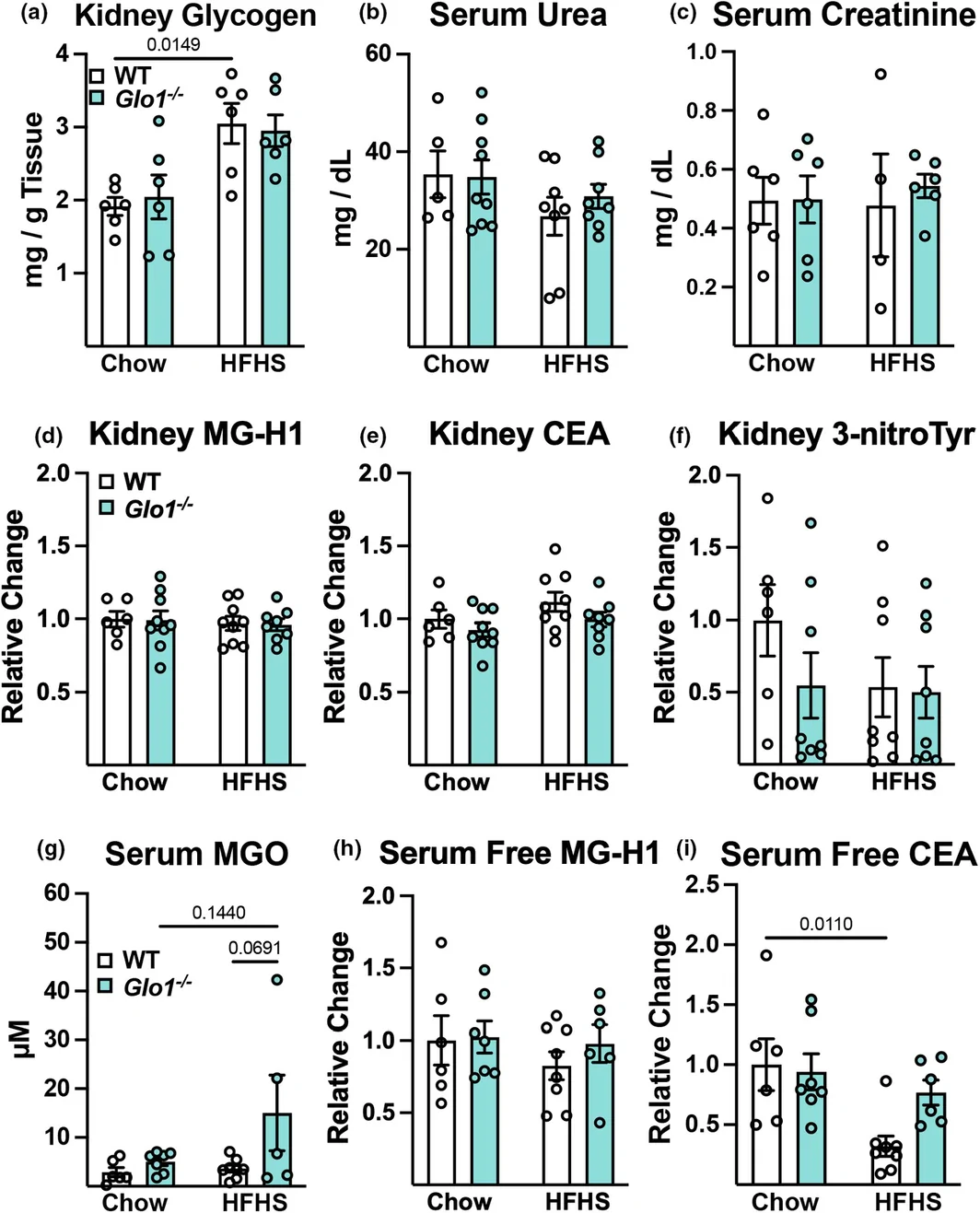
GLO1 knockout does not result in significant kidney pathologies. (a) Kidney glycogen is significantly elevated in WT HFHS‐fed mice, while no significant differences are observed in *Glo1* ^−/−^ mice. (b) Serum urea and (c) creatinine are unchanged in all cohorts. Kidney (d) MG‐H1, (e) CEA, and (f) 3‐nitroTyr are unchanged in all cohorts. (g) Serum MGO is modestly elevated in *Glo1* ^−/−^ mice. Chow‐fed WT (*N* = 7), chow‐fed *Glo1* ^*−/−*^ mice (*N* = 7), HFHS‐fed WT (*N* = 8), HFHS‐fed *Glo1* ^*−/−*^ mice (*N* = 6). The free MGO‐modified amino acids, (h) MG‐H1 and (i) CEA are unchanged as a result of *Glo1* status. Chow‐fed WT (*N* = 7), chow‐fed *Glo1* ^*−/−*^ mice (*N* = 5), *N* = 6 for HFHS‐fed cohorts. Statistical significance was determined via Two‐Way ANOVA.

Previous work using shRNA targeting *Glo1* showed spontaneous generation of pathologies consistent with diabetic nephropathy in mice (Giacco et al., 2014). We thus reasoned that 16 weeks of HFHS feeding would exacerbate this phenotype. To determine possible kidney injury, we first quantified kidney glycogen (Figure 6a). While a HFHS diet results in the accumulation of kidney glycogen, the absence of GLO1 does not alter these levels. We next quantified serum urea (Figure 6b) and creatinine (Figure 6c) as measures of kidney function and found no significant differences in any cohort. We also quantified renal MG‐H1 (Figure 6d), CEA (Figure 6e), and 3‐nitrotyrosine (Figure 6f), a PTM associated with oxidative stress that has been reported to be elevated in diabetic kidneys (Geoffrion et al., 2014; Giacco et al., 2014). These measurements reveal no significant differences resulting from either diet or *Glo1* ^−/−^.

Previous work has identified the kidney as a critical regulator of circulating MGO and MGO‐derived modifications (Lai et al., 2024). To interrogate this, we quantified serum MGO and circulating MGO‐derived free amino acids, MG‐H1 and CEA. While significance is not achieved, serum MGO is elevated in *Glo1* ^−/−^ HFHS‐fed mice, with no differences observed in the chow‐fed cohorts (Figure 6g). Despite this elevation in MGO, however, no significant differences were observed in either free MG‐H1 (Figure 6h) or CEA (Figure 6i). In fact, a significant diet‐induced decrease in CEA is observed in WT HFHS‐fed mice. Although other metabolites, notably carboxyethyl‐deoxyguanosine (CEdG), were not quantified here, our measurements of free MGO, protein‐bound MGO‐derived PTMs, and circulating free MGO‐derived PTM monomers provide a thorough readout of MGO and GLO1 function in diabetic kidney disease.

## 4 DISCUSSION

Although the role of GLO1 in the pathogenesis of diabetic kidney disease has been investigated, its contribution to MAFLD has remained poorly defined. A report by Spanos et al. observed reduced GLO1 expression in *Apoe* ^−/−^ mice, suggesting that GLO1 in the pathogenesis of MAFLD is likely (Spanos et al., 2018). Furthermore, using immunohistochemistry, reduced expression of GLO1 was observed in tissue biopsies from MAFLD patients; a quantitative analysis of GLO1 expression, however, was not performed (Spanos et al., 2018). These observations established an association between reduced GLO1 expression and MAFLD but did not address whether loss of GLO1 is causal. Our results demonstrate Glo1 ablation limits triglyceride accumulation during HFHS feeding, supporting a previously unrecognized role for GLO1 in regulating hepatic lipid metabolism. Interestingly, protection from hepatic steatosis occurred despite a modest worsening of glucose tolerance, suggesting hepatic lipid accumulation and systemic glycemic control are mechanistically distinct in the absence of GLO1. A caveat of the present study is that the dietary intervention was terminated after 16 weeks, a timepoint in which significant hepatic steatosis is evident but more advanced features of MAFLD (e.g. inflammation) have not developed. Thus, whether GLO1 influences disease progression at more advanced stages of MAFLD, including MASH, remains unknown and is a topic of future investigations.

Our data also challenges previous reports suggesting a predominant role for GLO1 in the pathogenesis of diabetes‐induced kidney injury (Geoffrion et al., 2014; Giacco et al., 2014; Rabbani & Thornalley, 2018), revealing that ablation of GLO1 alone is not sufficient to drive kidney pathology, consistent with Shumacher et al (Schumacher et al., 2018). Whereas Giacco et al. reported spontaneous diabetic nephropathy following shRNA‐mediated Glo1 knockdown (Giacco et al., 2014), Schumacher et al. found no evidence of worsened kidney pathology in *Glo1* ^−/−^ mice, even when challenged with streptozotocin treatment (Schumacher et al., 2018). Our findings independently support those of Schumacher et al., clearly demonstrating no impact on overall kidney health, regardless of diet or genotype (Schumacher et al., 2018). Importantly, however, our study did not evaluate additional modes of MGO regulation as these are thought to play a minor role in MGO detoxification. Whether these compensatory pathways become more important under conditions of chronic metabolic stress remains unknown; however, these data collectively indicate that loss of GLO1 alone is insufficient for the generation of diabetic nephropathy.

MGO has long been classified as a toxic by‐product of metabolism (Schalkwijk & Stehouwer, 2020); however, multiple studies have demonstrated that cytotoxic concentrations of MGO occur in the high μM—low mM range, depending on cell type (Galligan et al., 2018; Nokin et al., 2016, 2017). These concentrations far exceed those measured in vivo, supporting the hypothesis that MGO plays a larger role in metabolic homeostasis, rather than exclusively disease pathology (Galligan et al., 2018; Rabbani & Thornalley, 2014). Recently, Kulkarni et al. observed a protective role for MGO in cardiac ischemia–reperfusion injury (Kulkarni et al., 2020). A similar phenomenon was observed in cancer, where slight elevations in MGO were found to stimulate tumor growth, while high concentrations, exceeding those observed in vivo, were found to be cytotoxic (Nokin et al., 2017). These observations are consistent with the concept that MGO exhibits concentration‐dependent biological effects, functioning as both a metabolic signal and a cytotoxic metabolite depending on the cellular context. An additional consideration is that loss of GLO1 reduces glucose uptake and glycolytic flux, which we recently reported in cultured cells (Trujillo et al., 2025). This reduced flux would limit the amount of substrate available for MGO generation. If a similar reduction in glycolytic flux occurs in vivo, it may counteract the expected rise in MGO production, potentially explaining why steady‐state MGO and MGO‐derived PTMs remain largely unchanged in *Glo1* ^*−/−*^ mice. Finally, we have also reported that *Glo1* ^−/−^ fibroblasts fail to differentiate to mature adipocytes (Trujillo et al., 2025). Although a comprehensive evaluation of adipocyte biology was not performed here, loss of GLO1 had minimal effects on adipocyte size, epididymal fat mass, or whole‐body β‐oxidation, suggesting that the hepatic phenotype is unlikely to be secondary to gross alterations in adipose tissue function. Future investigations will be aimed at deciphering the tissue‐specific roles of GLO1 in whole‐body glucose tolerance and lipid metabolism.

## AUTHOR CONTRIBUTIONS

**Emely A. Hoffman:** Data curation; methodology. **Aiden M. Phoebe:** Data curation; formal analysis. **Marissa N. Trujillo:** Data curation; methodology. **Wei Chen Zhang:** Data curation; formal analysis. **Erin Q. Jennings:** Data curation; formal analysis. **Dominique O. Farrera:** Conceptualization. **David J. Orlicky:** Data curation; formal analysis. **Lauren N. Rutt:** Data curation; formal analysis. **Rebecca L. McCullough:** Funding acquisition; supervision. **Andrea A. Huacachino:** Data curation; formal analysis. **Mariola M. Marcinkiewicz:** Data curation; formal analysis. **Nathaniel W. Snyder:** Funding acquisition; supervision. **Kassandra R. Bruner:** Data curation; formal analysis. **Kimberly Bassoco Payan:** Data curation; formal analysis. **Diego Juan Federico Martinez:** Data curation; formal analysis. **Jennifer H. Stern:** Data curation; formal analysis; funding acquisition; supervision. **James J. Galligan:** Conceptualization; data curation; formal analysis; funding acquisition; investigation; methodology; project administration; supervision; visualization.

## FUNDING INFORMATION

Financial support was provided by National Institutes of Health Grants: R01 DK133196 for J.J.G.; T32 GM008804 for M.N.T.; R01 DK138011 and R35 GM156596 to N.W.S.; F31 AA031426 to L.N.R.; R01 AA030741 to R.L.M. The content is solely the responsibility of the authors and does not necessarily represent the official views of the National Institutes of Health.

## CONFLICT OF INTEREST STATEMENT

The authors declare no competing interests.

## ETHICS STATEMENT

All animal use was approved by the Institutional Animal Care and Use Committee of the University of Arizona and adhered to the NIH Guide for the Care and Use of Laboratory Animals under protocol number 18‐445.

## Supporting information


**Figure S1.** GLO1 does not alter hepatic insulin signaling. (a) Immunoblotting was conducted against phospho‐AKT Ser473 (pAKT(S473)), total AKT, phospho‐insulin receptor (pINS‐R), total insulin receptor (INS‐R), and actin. (b) Blot densitometry was performed with ImageJ. Phospho‐ and total protein levels were each normalized to their own actin. Plotted values represent the phospho/total‐protein ratio, expressed relative to WT chow‐fed mice. Blots were performed in triplicate (*N* = 3). Data are shown as the mean +/− SEM, with statistical significance was determined via Two‐Way ANOVA.
**Figure S2.** Gross tissue mass. (a) eWAT, (b) Liver, and (c) kidney masses in each cohort. Chow‐fed WT (*N* = 6), chow‐fed Glo1−/− mice (*N* = 9), HFHS‐fed WT mice (*N* = 9), HFHS fed Glo1−/− mice (*N* = 8). Statistical significance was determined via Two‐Way ANOVA.

## Data Availability

All data associated with this study is available from the corresponding author upon reasonable request.
