# Supplementary material for: Glyoxalase 1 loss reduces fatty liver but impairs glucose handling in male mice
Source: Physiol Rep. 2026 Sep 14;14(17):e71106. doi: 10.14814/phy2.71106 (PMC13575502; doi:10.14814/phy2.71106)

# Glyoxalase 1 Loss Reduces Fatty Liver but Impairs Glucose Handling in Male Mice

Emely A. Hoffman[1], Aiden M. Phoebe[1], Marissa N. Trujillo[1], Wei Chen Zhang[1], Erin Q. Jennings[1], Dominique O. Farrera[1], David J. Orlicky[2], Lauren N. Rutt[3], Rebecca L. McCullough[3], Andrea A. Huacachino[4], Mariola M. Marcinkiewicz[4], Nathaniel W. Snyder[4], Kassandra R. Bruner[5], Kimberly Bassoco Payan[5], Diego Juan Federico Martinez[5], Jennifer H. Stern[5], and James J. Galligan[1]*

[1]Department of Pharmacology and Toxicology, College of Pharmacy, University of Arizona, Tucson, AZ

[2]Department of Pathology, University of Colorado Anschutz Medical Campus, Aurora, CO

[3]Department of Pharmaceutical Sciences, The Skaggs School of Pharmacy and Pharmaceutical Sciences, University of Colorado Anschutz Medical Campus, Aurora, CO

[4]Aging & Cardiovascular Discovery Center, Department of Cardiovascular Sciences, Lewis Katz School of Medicine at Temple University, Philadelphia, PA

[5]Division of Endocrinology, Department of Medicine, College of Medicine, University of Arizona, Tucson, AZ

*To whom correspondence should be addressed:

James J. Galligan

1703 E. Mabel St.

Room 205

Tucson, AZ 85721

Phone: 520-621-6015

E-mail: jgalligan@pharmacy.arizona.edu

**Author Contributions:** E.A.H., D.O.F, E.Q.J., and J.J.G. designed research; E.A.H., A.M.P., M.N.T. D.O.F., D.J.O., W.C.Z., K.R.B., K.B.P., D.J.F.M., L.N.R., A.A.H., and M.M.M. performed research; A.M.P., E.A.H., N.W.S., and J.J.G. analyzed data; and A.M.P., E.A.H. and J.J.G. wrote the paper; J.H.S., N.W.S., R.L.M., and J.J.G. provided the funding.

**Competing Interest Statement:** The authors declare no competing interests.

**Classification:** Biological Sciences, Biochemistry

**Keywords:** methylglyoxal, glycolysis, glycation, glyoxalase, MAFLD

**Supplemental Figure Legends.**

**Supplemental Figure 1. GLO1 does not alter hepatic insulin signaling.** (a) Immunoblotting was conducted against phospho-AKT Ser473 (pAKT(S473)), total AKT, phospho-insulin receptor (pINS-R), total insulin receptor (INS-R), and actin. (b) Blot densitometry was performed with ImageJ. Phospho- and total protein levels were each normalized to their own actin. Plotted values represent the phospho/total-protein ratio, expressed relative to WT chow-fed mice. Blots were performed in triplicate (N=3). Data are shown as the mean +/- SEM, with statistical significance was determined via Two-Way ANOVA.

**Supplemental Figure 2. Gross tissue mass.** (a) eWAT, (b) Liver, and (c) kidney masses in each cohort. Chow-fed WT (N = 6), chow-fed *Glo1*$^{-/-}$ mice (N = 9), HFHS-fed WT mice (N = 9), HFHS-fed *Glo1*$^{-/-}$ mice (N = 8). Statistical significance was determined via Two-Way ANOVA.

**Supplemental Figure 1.**

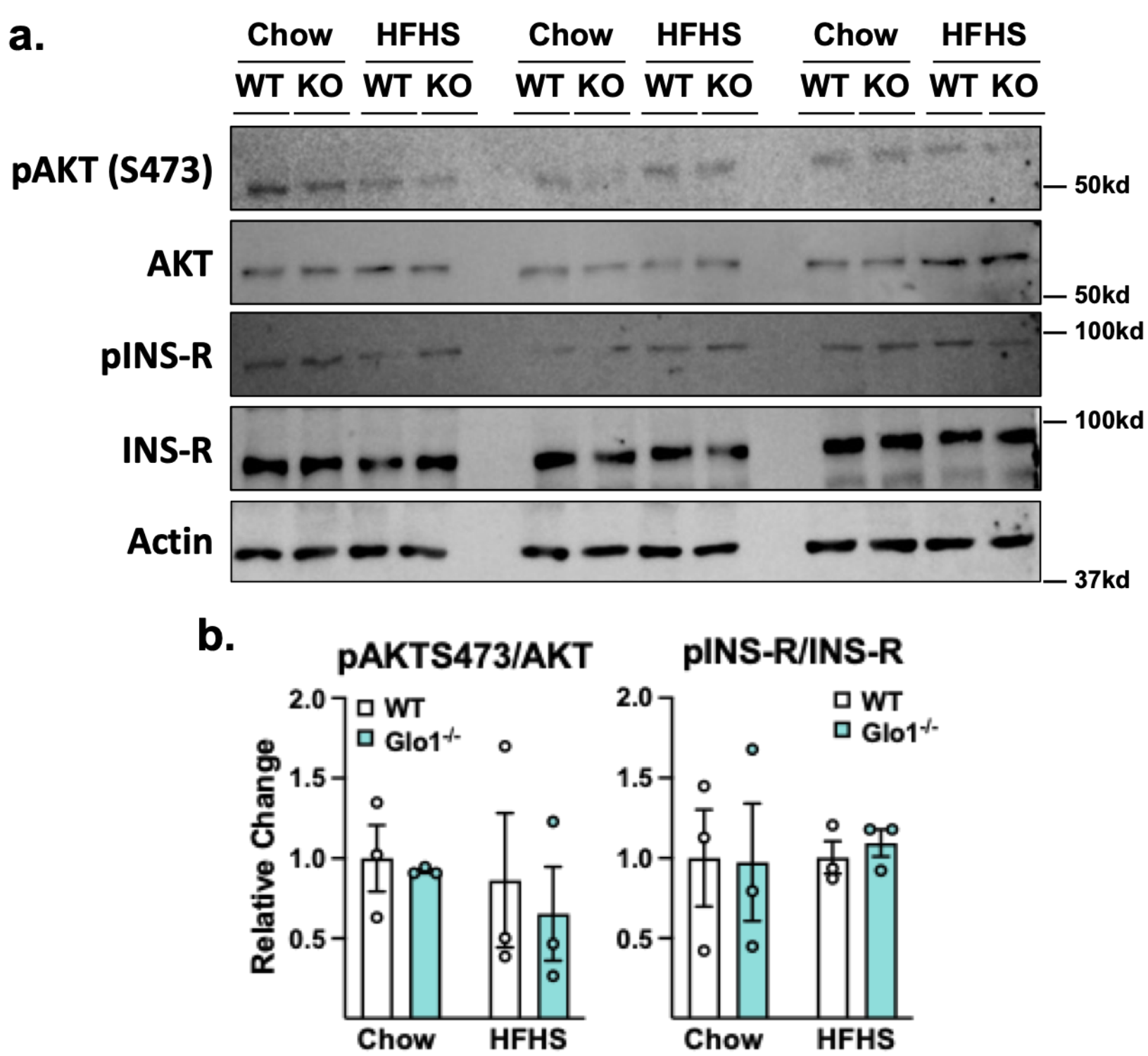

**Supplemental Figure 2.**

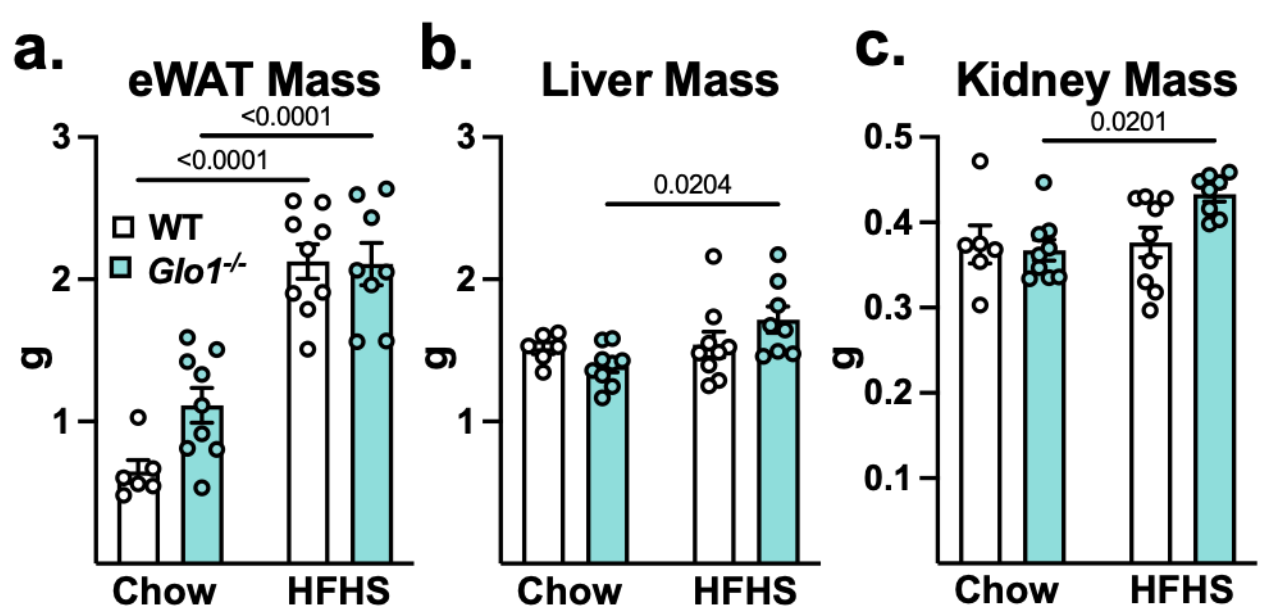

Supplement: Supplementary file 1 [file PHY2-14-e71106-s001.pdf]
